# Characterization and sequence prediction of structural variations in *α*-helix

**DOI:** 10.1186/1471-2105-12-S1-S20

**Published:** 2011-02-15

**Authors:** Ashish V Tendulkar, Pramod P Wangikar

**Affiliations:** 1 Department of Computer Science and Engineering, Indian Institute of Technology Madras, Chennai-600 036, India; 2 Department of Computer Science and Engineering, Indian Institute of Technology Bombay, Powai, Mumbai-400 076, India; 3 Department of Chemical Engineering, Indian Institute of Technology Bombay, Mumbai-400 076, India

## Abstract

**Background:**

The structure conservation in various *α*-helix subclasses reveals the sequence and context dependent factors causing distortions in the *α*-helix. The sequence-structure relationship in these subclasses can be used to predict structural variations in *α*-helix purely based on its sequence. We train support vector machine(SVM) with dot product kernel function to discriminate between regular *α*-helix and non-regular *α*-helices purely based on the sequences, which are represented with various overall and position specific propensities of amino acids.

**Results:**

We characterize the structural distortions in five *α*-helix subclasses. The sequence structure correlation in the subclasses reveals that the increased propensity of proline, histidine, serine, aspartic acid and aromatic amino acids are responsible for the distortions in regular *α*-helix. The N-terminus of regular *α*-helix prefers neutral and acidic polar amino acids, while the C-terminus prefers basic polar amino acid. Proline is preferred in the first turn of regular *α*-helix , while it is preferred to produce kinked and curved subclasses. The SVM discriminates between regular *α*-helix and the rest with precision of 80.97% and recall of 88.05%.

**Conclusions:**

The correlation between structural variation in helices and their sequences is manifested by the performance of SVM based on sequence features. The results presented here are useful for computational design of helices. The results are also useful for prediction of structural perturbations in helix sequence purely based on its sequence.

## Background

The *α*-helix is the most important structural element in proteins, first described by Pauling in 1951 [1]. The helices in protein can be classified as left handed and right handed helix based on their handedness. The right handed *α*-helices are found more frequently in the proteins than their left handed counterparts [1]. The right handed *α*-helix is a regular structure with backbone torsion angles of *φ* = –63 and *ψ* = –43 [1-3].

Although *α*-helix is regular in nature, it shows significant imperfection in its structure due to a variety of reasons. For example, proline residue beyond first turn in *α*-helix causes a kink in its structure [3]. The perturbations in the helix geometry give rise to different subclasses of *α*-helix. The three types of helix subclasses are reported in the literature: linear, curved and kinked [2,3]. It is well known that the structural variations in *α*-helix are encoded in its sequence. The preferences of different amino acids either for or against being in *α*-helix in general or at any specific position in it are reported in literature [1,2,4,5]. These sequence based features form the basis of prediction of helix from amino acid sequence of the protein [6]. Given that the methods for prediction of helix from amino acid sequence have matured, we need to step further up and predict finer structural variations in the helix based on its sequence.

In our earlier work, we had performed Gaussian mixture modeling of octapeptide helix conformations based on their geometric invariant structure descriptors. It resulted in 11 subclasses of helix, which represent the structural variations of one form or the other in the helix. We first characterize the form of structural variation in *α*-helix in the subclasses and remove the redundant subclasses. We then analyze sequence-structure correlation in the subclasses. We train support vector machine [7](SVM) to predict structural variations in *α*-helix based on the sequence. Support Vector Machines(SVMs) are a class of supervised learning algorithms based on statistical learning theory [7]. Given the set of positive and negative training examples, SVMs learn a linear decision boundary to discriminate between the two classes. Thus, the linear classifier obtained by SVMs is known to exhibit excellent generalization performance [7]. SVMs have been applied extensively in micro-array data analysis [8], prediction of sub-cellular location of proteins [9] and in web mining [10]. We achieve precision of 80.97% and recall of 88.05% in discriminating regular *α*-helix sequences from the other helix sequences containing structural variations.

## Results

The input dataset for Gaussian mixture modeling contains approximately 0.4 million octapeptide helices drawn from ASTRAL 95 dataset (version 1.67) [11] based on the criteria defined in [12]. The geometry of the helices was approximated in terms of their *C_α_* geometry. The structure of an individual helix was described using a set of 29 geometric invariants described in [13]. The PCA reveals that the first 6 PCs explains 80% variance in the dataset. Thus, the structural space of the local conformations is described with 6 PCs.

### Finer subclasses of helix

The Gaussian mixture modeling results in 11 *α*-helix subclasses with skewed mixing proportions. The analysis of the subclasses reveals five important helix subclasses-(i)right handed regular *α*-helix [1,3], (ii) extended helix, (iii) c-cap helix, (iv) kinked helix [3], and (v) curved helix [2]. We find that the regular *α*-helix is the most dominant subclass having as much as 76% mixing proportion. The extended helix and c-cap helix are the second largest subclasses with mixing proportion of 5% each. The kinked helix subclass have 2% mixing proportion, while the curved helix subclass have the least mixing proportion of 1%.

Note that the remaining six subclasses represent the same structural variation in helix at different locations due to the overlap of seven amino acids between the neighboring octapeptides in the input dataset. For example, the subclasses 4, 6, 7, and 9 represent the same kink variation at the fourth, fifth, sixth and seventh position respectively. We select subclass 6 to represent kinked variations in *α*-helix.

### Structural variations in Helix subclasses

#### Gross structural variations

We analyzed the gross geometric invariants such as *d*_18_, and *A*_158_ (Table 1). The analysis reveals that the regular *α* helix subclass has the minimal standard deviation compared to all other subclasses. It signifies strong regularity in the nature of helix as against the subclasses having higher standard deviations for the gross geometric properties. The larger *d*_18_ corresponds to an extended helix structure. Based on *d*_18_, the n-cap subclass is the most extended helical structure followed by the kinked helix subclass. The curved helix is the most compact helix subclass, while the c-cap is in between regular *α*-helix and the curved helix in terms of compactness. The decrease in *d*_18_ along with the increase in *A*_1,5,8_ denotes curved nature of helix. The decrease in both the geometric invariants denotes more compact helix, while the increase in both denotes an extended helix. Thus, c-cap and curved helix subclasses contain a curve in their structures while the n-cap and kinked helix subclasses are extended structures.

**Table 1:** Mean and standard deviation of gross structure descriptors for helix subclasses

Subclass	*d*_18_	*A*_158_
Regular *α*-helix	10.64+/-0.34	10.40+/-1.50
Extended helix	11.51+/-0.59	10.66+/-4.04
Helix with c-cap	10.35+/-0.65	15.21+/-2.32
Kinked helix	11.39+/-0.82	18.52+/-4.25
Curved Helix	8.64+/-1.58	12.12+/-6.23

(i) *d*_18_ denotes distance between  of the octapeptide helix conformations. (ii) *A*_158_ denotes area of triangle formed by .

#### Finer structural variations

**Figure 1:**
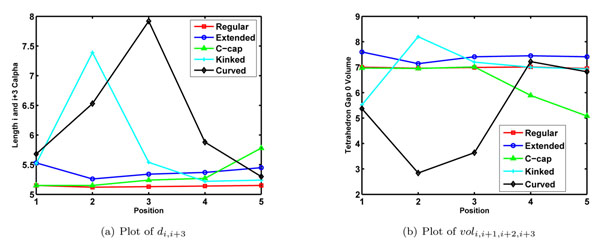
**Plots for finer geometric invariants for different helix subclasses**. The octapeptide helical structures are divided into five overlapping tetrahedrons consisting of . For each of the tetrahedra, we plot: (a) *d_i_*_,_*_i_*_+3_ denotes distance between the first and the last *C_α_s* in tetrahedron (b) *vol_i_*_,_*_i_*_+1,_*_i_*_+2,_*_i_*_+3_ represent volume of the tetrahedron.

The octapeptide helical conformations are divided into five overlapping tetrahedrons formed by the four consecutive . Thus, the five tetrahedron represent finer structural characteristics of the *α*-helix structure. The tetrahedrons are in turn described using *d_i_*_,_*_i+_*_3_ and *vol_i_*_,_*_i+_*_1,_*_i+_*_2,_*_i+_*_3_ geometric invariants. The geometric invariants are plotted in (fig. 1a).

The regular *α*-helix appears as a straight line in both the plots. This implies that all the five tetrahedrons of the regular *α*-helix subclass are identical in terms of their geometries. The rest of the other subclasses show structural perturbations at different locations. The tetrahedrons corresponding to the structural perturbations can be classified with respect to regular tetrahedron corresponding to the regular *α*-helix: (i)Extended tetrahedrons which have more *d_i_*_,_*_i_*_+3_ as well as *vol_i_*_,_*_i_*_+1,_*_i_*_+2,_*_i_*_+3_ than their regular counterpart, and (ii)Compact tetrahedrons which have more *d_i_*_,_*_i_*_+3_ and lesser *vol_i_*_,_*_i_*_+1,_*_i_*_+2,_*_i_*_+3_ than their regular counterpart. Moreover, the sign of *vol_i_*_,_*_i_*_+1,_*_i_*_+2,_*_i_*_+4_ characterizes handedness of the helix. The positive values of *vol_i_*_,_*_i_*_+1,_*_i_*_+2,_*_i_*_+4_ indicates right handed helix, whereas the negative values indicates left handed helix. The positive values of all the tetrahedrons implies that all the subclasses contains right handed helices.

All the tetrahedrons in the extended helix subclass are extended in nature with a little variation. The first tetrahedron is more extended, while the second tetrahedron is less extended than the rest of the tetrahedra. It implies that the extended helix subclass is almost a regular helix with more *d_i_*_,_*_i_*_+3_ than the regular helix subclass throughout its structure. The c-cap subclass contains regular tetrahedrons in its N-terminus region, while compact tetrahedrons in the C-terminus region. It implies that the c-cap subclass has a compact structure in its c-terminus region. Moreover, the structural stretch of first five residues in c-cap subclass is structurally similar to regular helix subclass. The kinked helix subclass contains mix of all types of tetrahedrons. The kinked helices appears to have a compact structure in N-terminus followed by a kink in the middle region and approximately regular structure in its C-terminus region. The curved helix subclass, on the other hand, appears to be a compact structure with curving middle region followed a slightly extended structure in region prior C-terminus region. The curved helix subclass ends with a slightly compact structure than the regular one. Moreover, the tetrahedrons at the either ends of curved helix are similar to that of kinked helix. This implies that the kinked and curved helix subclass have similar structure in N and C terminus region.

### Sequence variations in helix subclasses

**Figure 2:**
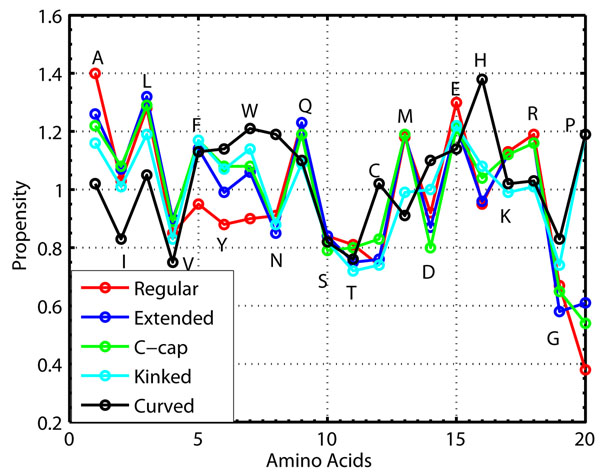
**Overall amino acid propensities for different helix subclasses.** Overall amino acid propensities for different helix subclasses.

The sequence properties such as overall and position specific amino acid propensities are calculated for the subclasses using variable length helices constructed using within subclass merging. The distribution of helices in the subclasses by their lengths is shown in Table 2. The overall amino acid propensities is shown in fig. 2. The list of favorable amino acids at each position in the helix subclasses is given in Table 3, 4 and 5.

**Table 2:** Distribution of helices by their lengths

Subclass	*l*=8	8 <*l* ≤ 15	15 <*l*
Regular *α*-helix	14.41%	59.60%	25.98%
Extended helix	72.72%	27.24%	0.02%
Helix with c-cap	79.03%	20.95%	0.01%
Kinked helix	99.31%	0.69%	0%
Curved Helix	93.02%	6.98%	0%

The table shows the distribution of helices in different subclasses by their lengths: (i) *l*=8 contains helices having length equal to 8 (ii) 8 <*l* ≤ 15 contains helices having length between 9 and 15, and (iii) 15 <*l* contains helices having length greater than 15.

**Table 3:** Preferred Amino Acids at N terminus of helix

Subclass	*N*_1_	*N*_2_	*N*_3_	*N*_4_
Regular *α*-helix	S/T/D/N	P	E	E/Q
Extended Helix	D/N	P/A/W/E/L	E/A/Q	E/L/F/Q
Helix with c-cap	D/E/A	Q/A/E	E/W/A/L	L/F/I/M
Kinked helix	P/E	E/P/D	N/H/Y/F	W/A/L/F
Curved Helix	Y/F/W	E/K/R	H/N/Q/K/E	N/D/H/C/G

We have listed the most preferred amino acids at N-terminus positions in the helix. At each position, the amino acids are arranged in descending order of their propensities. The positions in the N-terminus are denoted by *N*_1_ to *N*_4_ from left to right.

**Table 4:** Preferred Amino Acids at C terminus of helix

Subclass	*C*_4_	*C*_3_	*C*_2_	*C*_1_
Regular *α*-helix	A/I/L	K/A/R/E/Q	L/A/Q/K/R	G
Extended Helix	L/M/A	L/Q/K	L/Q/K	L/F/Y/Q/M
Helix with c-cap	L/A/R/M	K/ R/ E/ Q/ L	H/ Y/ F	G
Kinked helix	P	E/Q/W	L/I/F/Y/V	L/M/I/A
Curved Helix	P	P/E	H/P/W	F/Y/L/W/A

We have listed the most preferred amino acids at C-terminus positions in the helix. At each position, the amino acids are arranged in descending order of their propensities. The position in the C-terminus are denoted by *C*_1_ to *C*_4_ from right to left.

**Table 5:** Preferred Amino Acids in the middle of helix

Subclass	*M*_1_	*M*_2_	*M*_3_	*M*_4_
Regular *α*-helix	I/L/A/M/F	A/K/R/Q/E	A/R/K/Q/E	L/M/A
Extended Helix	L/M/A	L/A	Q/K/R/L/M	L/F
Helix with c-cap	L/A/M/R	K/L/A/Q/E	H/F/Y/N	G/F/L/Y/H

The propensity for the middle four positions are calculated for the sequences containing minimum 12 amino acids. The first middle position represent the fifth position in the sequence from the N-terminus end. The amino acids at each position are arranged in descending order of their propensities. The kinked and curved helix do not have sequences more than length 8 and do not figure out in this list.

#### Overall amino acid propensity analysis

The overall amino acid propensities for the helix subclasses reveals preferences of particular amino acids over other in the respective subclasses(Fig. 2).

The overall propensities of the aliphatic amino acids reveals that *ala* and *leu* are the most favorable aliphatic amino acids to form all the helix subclasses. We also observe that *ile* is a favorable amino acid to form all the helix subclasses except the curved one. *Gly* and *val* remains less preferred aliphatic amino acids in all the helix subclasses. *Pro*, a well-known helix breaker, is equally preferred in kinked and curved helix formation. The decrease in *ala* and *leu* propensity in kinked and curved helix is compensated by the increase in *pro* propensity. Thus, the aliphatic amino acids like *ala* and *leu* are more favorable to form regular helices, while pro is more favorable to form less regular helices such as kinked and curved ones. The aromatic amino acids have the least propensity values for regular *α*-helix. However, the aromatic amino acids are more favorable in the rest of helix subclasses. We observe that *phe* is equally favorable in all the helix subclasses except the regular one, *tyr* is slightly more favorable in kinked and curved helices than the c-cap, and *trp* is the most favorable aromatic amino acid in curved helices. This implies that the aromatic amino acids are instrumental for distortion in regular helix along with *pro*.

Out of the neutral polar amino acids, *asn*, *ser* and *thr* are less preferred amino acids in all the helix subclasses except the curved one. The curved helix subclass seems to prefer *asn* as one of the most favorable amino acid. *Gln* is preferred amino acid in all the helix subclasses. The *gln* propensity also follows the trend of *ala* and *leu* propensities and decreases in the kinked and curved helix subclasses. The analysis of overall amino acid propensity of charged amino acids reveals that *asp* has more higher propensity in curved and kinked helices than the rest of the subclasses. It also reveals that *his* is favorable amino acid in c-cap, kinked and curved helix subclasses. The amino acids like *glu*, *arg* and *lys* are more favorable in the regular *α* helix, n-cap and c-cap subclasses than the kinked and the curved subclasses. Thus, the drop in the propensities of *glu*, *arg* and *lys* seems to be compensated with increase in propensities of *asp* and *his* in kinked and curved helix subclasses.

The overall amino acid propensity of *met* reveals that the amino acid is equally favorable in regular, n-cap and c-cap subclasses and less favorable in kinked and curved helix subclasses. The overall amino acid propensity of *cys* reveals that the amino acid is less favorable in all the helix subclasses except the curved helix subclass.

#### Position specific amino acid propensities

The position specific amino acid propensities are calculated for N and C terminus of helices. For the helices with length greater 12, we also computed the position specific amino acid propensities for the middle region. Note here that the last position in n-terminus *N*_4_ and the first position in c-terminus *C*_4_ need not be adjacent to each other in regular, n-cap and c-cap subclasses. The analysis of position specific amino acid propensities reveals distinct position wise amino acid preferences in different helix subclasses.

The regular *α*-helix subclass appears to prefer small polar amino acids such as *ser*, *thr*, *asn* and *asp* at *N*_1_ position. It strongly prefers *pro* at the *N*_2_ position and is strongly avoided at the rest of the positions. It strongly prefers *glu* at the *N*_3_ position. It strongly prefers *glu* and its neutral derivative *gln* at *N*_4_ position. It strongly prefers aliphatic amino acids such as *ala*, *ile* and *leu* at *C*_4_ position. It strongly prefers polar amino acids such as *lys*, *arg*, *glu*, and *gln* at *C*_3_ position. It also prefers *ala* at *C*_3_ position. It strongly prefers aliphatic amino acids such as *leu* and *ala* along with positively charged amino acids such as *lys* and *arg* and polar neutral amino acid such as *gln* at *C*_2_ position. At the *C*_1_ position, it strongly prefers *gly*, indicating a possible loop following helix.

The extended helix subclass appears to prefer charged amino acid *asp* and its neutral derivative *asn* at *N*_1_ position. It mostly refers hydrophobic amino acids such as *pro*, *ala*, *trp*, *leu* at *N*_2_ position. However, it also prefers *glu* at *N*_2_ position, which is a charged polar amino acid. It prefers *glu*, *ala*, and *gln* at *N*_3_ position. It prefers large amino acids such as *glu*, *gln*, *leu* and *phe* at *N*_4_ position. It strongly prefers hydrophobic amino acids such as *leu*, *ala* and *met* at *C*_4_ position. It appears that the amino acid preferences for *C*_3_ and *C*_2_ positions are identical. The amino acids such as *leu*, *gln* and *lys* are preferred in these positions. Finally, the *C*_1_ position of the extended helix subclass prefers a mix of polar and apolar amino acids such as *leu*, *phe*, *tyr*, *gln* and *met*.

The c-cap helix subclass appears to prefer charged amino acids such as *asp* and *glu* as well as *ala*, which is a small hydrophobic amino acid at *N*_1_ position. It prefers *gln*, *glu* and *ala* at *N*_2_ position. It prefers *glu* along with hydrophobic amino acids such as *trp*, *ala* and *leu* at *N*_3_ position. At *N*_4_ position, it strongly prefers large hydrophobic amino acids such as *leu*, *ile* , *phe* and *met.* It prefers mostly hydrophobic amino acids such as *leu*, *ala* and *met* along with *arg*, which is a positively charged amino acid at *C*_4_ position. It strongly prefers polar amino acids at *C*_3_ and *C*_2_ positions. The *C*_3_ position prefers *lys*, *arg*, *glu* and *gln* along with *leu*, which is a strongly hydrophobic amino acid, while the *C*_2_ position prefers *his* and *tyr*. At *C*_1_ position, it prefers *gly*.

The kink helix subclass prefers *pro* and *glu* at *N*_1_ position. It prefers *pro* along with negatively charged amino acids( *asp* and *glu* ) at *N*_2_ position. At *N*_3_ positions, it mostly prefers hydrophilic amino acids such as *his*, *asn* and *tyr* along with *phe*, which is an aliphatic amino acid. At *N*_4_ position, it predominantly prefers hydrophobic amino acids such as *trp*, *ala*, *leu* and *phe.* At *N*_5_ position, it strongly prefer *pro*, which is the cause of a kink at this position. It prefers negatively charged amino acid *glu* and its neutral derivative *gln* along with *trp* at *N*_6_ position. The last two positions, *C*_2_ and *C*_1_ strongly prefers aliphatic amino acids such as *ala*, *ile*, *leu* and *val*, with exception of *tyr* at *C*_2_ position and *met* at *C*_1_ position. The curved helix prefers aromatic amino acids at *N*_1_ position followed by a strongly polar amino acids at *N*_2_, *N*_3_ and *N*_4_ positions. It also prefers *cys* and *gly* at *N*_4_ positions. It strongly prefers *pro* at *C*_4_, *C*_3_ and *C*_2_ positions. In addition, it prefers *glu* at *C*_3_, *his* and *trp* at *C*_2_ positions. The *C*_1_ position of the curved helix prefers a mix of aliphatic and aromatic amino acids. The aliphatic amino acids preferred at *C*_1_ are *phe*, *leu*, and *ala*, while the preferred aromatic amino acids include *trp*, and *tyr*.

The analysis of amino acid propensities at the middle positions in *α*-helix provides clues about sequence nature of the middle stretch in different subclasses. The regular *α* helix appears to prefer hydrophobic amino acids at *M*_1_ and *M*_4_ positions. It prefers hydrophilic amino acids such as *arg*, *lys*, *glu* and *gln* at *M*_2_ and *M*_3_ positions along with *ala.* The extended helix appears to prefer hydrophobic amino acids such as *ala*, *met*, *leu* and *phe* at *M*_1_, *M*_2_ and *M*_4_ position, while the *M*_3_ prefers hydrophilic amino acids such as *gln*, *lys*, and *arg* along with hydrophobic amino acids such as *met* and *leu.* The c-cap helix prefers strong hydrophobic amino acids such as *ala*, *met* and *leu* at *M*_1_ position along with *arg.* It prefers a mix of hydrophilic and hydrophobic amino acid residues at *M*_2_ position. It prefers *his* along with aromatic amino acids such as *phe* and *tyr* at *M*_3_ and *M*_4_ position. It prefers *asn* at *M*_3_ position, while *gly* at *M*_4_ position.

### Sequence based prediction of structural variations

We train SVM to discriminate between regular *α*-helices and the helices containing structural variations in form of kink, curve or capping. The training examples for SVM consists of helices having maximum length of 15 selected from the collection of variable length helices formed by across subclass merging. We select those helices which entirely belong to a single subclass. The helices containing more than one subclasses are not considered. Thus, we extract in all 28223 helices to form the training examples. Out of these examples, we have 17532 positive examples corresponding to regular *α*-helix and 10961 negative examples corresponding to the rest of the subclasses. The SVM is trained using 70% of the examples and tested with the remaining 30% of examples.

The sequences in the training examples are represented with a set of features calculated by dividing sequence into different subsequences. The first four amino acids form N-terminus subsequence, while the last four amino acids forms C-terminus subsequence. The middle subsequence corresponding to the helices having length 8 is formed with the amino acids between position 4 to 6. The middle subsequences in the sequences having length greater than 8 is formed with amino acids between position 4 to the start of C-terminus position. We calculate overall amino acid propensities for 20 amino acids for each of these subsequences. In addition, we also calculate the position specific propensities for 20 amino acids in N-terminus and C-terminus subsequences. Note that we do not calculate position specific propensities for the middle subsequence as majority of sequences in negative examples are of size 8. We also calculate overall and amino acid propensities corresponding to sequences of 4 amino acids prior and after the helices. The features corresponding to sequences prior and after the helices encode the environment and structural context around the helix. Thus, we have in all 440 features to represent each single sequence in the data. The analysis of the weights of the features learnt by the SVM during the training process reveals differences between regular and other subclasses in terms of the sequence properties. The topmost positive features corresponds to the overall amino acid propensities of strong aliphatic amino acids such as *leu*, *ile* and *ala*. It implies that the regular *α*-helix have higher overall propensities for *leu*, *ile* and *ala* than the other subclasses. The feature corresponding to position specific propensity of *gly* at the last position of the regular *α*-helices also receives strong positive weight. It implies that *gly* is more preferred amino acid at the end of regular *α* helix than the other subclasses. The feature corresponding to the overall propensity of proline in the sequence is assigned a strong negative weight. It implies that the higher proline propensity in a sequence is a good indicator of structural variation in regular *α*-helix. The features corresponding to overall propensity of *asp* and *gly* are also assigned strong negative weights along with position specific propensities of *pro* at *C*_2_ and *C*_3_ positions. We also find that the weights assigned to different features corresponding to the overall and position specific amino acid propensities of the sequence are in accordance with the patterns described in the earlier sections. The features corresponding to overall amino acid propensities of the neutral and acidic polar amino acid in the N-terminus receive strong positive weights, while those corresponding to basic polar amino acids in the C-terminus receive strong positive weights. The features corresponding to overall amino acid propensities of strong and large hydrophobic amino acids in the middle position receive strong positive weights. It broadly implies that the regular *α*-helix contains neutral and acidic polar amino acids at its N-terminus followed by large hydrophobic amino acids and polar basic amino acids in the C-terminus. The overall and position specific amino acid propensities of the 4 amino acids prior to N-terminus suggest that *gly* is most likely to be present prior to the N-terminus of regular *α*-helices, while the aliphatic amino acids are strongly disfavored in this region. The overall and position specific amino acid propensities of the 4 amino acids beyond C-terminus suggest that *pro* is most likely to be present beyond C-terminus of regular *α*-helix.

We use 10-fold cross-validation to measure the performance of SVM. The SVM achieves precision of 80.97% and recall of 88.05% on the test examples leading to F1 score of 84.51%. Note that these results are obtained on the dataset containing approximately 62% regular and 38% non-regular helices with 10-fold cross-validation.

## Discussion

The prediction of structural variations in the helices based on their sequences using SVM with an accuracy of 84.51% is the novel feature of the work. The correlation between structural variation in helices and their sequences is manifested by the performance of SVM based on sequence features.

We first obtain subclasses of *α*-helix using Gaussian mixture modeling of octapeptide helical structures represented with geometric invariants. The subclasses are further curated to retain five distinct subclasses denoting regular helix, extended helix, helix with c-cap, c-cap kinked helix, and curved helix. The subclasses shows distinct overall structural characteristics (Table 1 ), which help in understanding compactness and extendedness of the overall helix geometry. The finer geometric invariants pinpoint the exact location of structural variations in helix subclasses( Fig 1).

The within subclass merging of the neighboring octapeptides ensures that we have complete helical stretch belonging to a particular subclass for establishing sequence structure correlation in it. This is extremely important in analyzing the position specific amino acid propensity as the extracted helices represent true positions in the sequence as against the octapeptide helices. Thus, our method provides structure based unbiased method to extract helical stretches from protein belonging to a particular subclass. We found that the subclasses have different overall and position specific amino acid properties. The regular *α*-helix subclass prefers amino acids like *ala*, *leu*, *ile*, *glu*, *gln*, *arg*, *met*, *and lys*, which are considered to be good helix formers [1]. The other subclasses show increase in overall propensities of *pro*, *his*, *asp*, *cys* and aromatic residues. We detect highest position specific *pro* propensities in subclasses. Thus, it appears that the increase in *his*, *cys*, *asp*, *pro* and aromatic amino acids are instrumental in causing structural perturbations in *α*-helices.

The across subclass merging of neighboring octapeptides provides structure based unbiased method to extract complete helices from the proteins. The extracted helices contain either a single subclass or more than one subclasses in them. We choose helices completely belonging to a single subclass for training SVM to discriminate between regular *α*-helix and the other subclasses. We divide the sequences in N-terminus, C-terminus and middle subsequences to capture the context in form of sequences properties in these subsequences. The context around the helices is captured in form of sequence of 4 amino acids prior to N-terminus and beyond C-terminus. The analysis of model learnt by SVM reveals that the structural variation in helices are result of the sequence variations and the structural context.

The results presented here are useful for computational design of helices. The results are also useful for prediction of structural perturbations in helix purely based on its sequence.

## Methods

### Structural characterization of helix subclasses

In our earlier work, we performed fine grain classification of helices in proteins into its subclasses using Gaussian mixture modeling [12]. We first extract octapeptide helical local conformations from the proteins in ASTRAL 95 dataset version(1.67) [11] as described in [12]. The helices are described using geometric invariant structure properties such as edge, perimeter, volume, area of triangle etc. [13]. The geometric invariants are then normalized to mean-centric, unity standard deviation values and subjected to principal component analysis(PCA) [14]. Thus, we transform the helical structures in principal component(PC) space bounded by the first *s* significant PCs. The structural space of helices is modeled as a mixture of *k* Gaussians, which one to one correspond to *k* subclasses. The parameters of the mixture are estimated using Expectation Maximization(EM) algorithm [15]. Thus, we obtain *k* subclasses of *α*-helix. The input helices are assigned to one of the *k* subclasses based on the scoring scheme described in [12]. We then characterize the form of structural variations described by each subclass based on their geometric invariants.

### Formation of longer helices

The octapeptide helical conformations in our dataset represent subpart of the actual helices in protein. The actual helices in the proteins can be reconstructed by merging the neighboring helices *h_i_* and *h_j_* in protein *P*. *h_i_* and *h_j_* share an overlap of seven amino acids between them. Further, *h_j_* is said to *follow h_i_* when the first seven residues in *h_j_* share an overlap with the last seven residues in *h_i_*. We can then merge *h_i_* and *h_j_* to form a helix having length one more than that of *h_i_*. Thus, the neighboring helices are merged to form actual helices of variable lengths for further sequence related analysis.

We use two types of merging: (i) within subclass and (ii) across subclass based on the merging criteria while forming longer helices. In the within subclass merging, the neighboring helices *h_i_* and *h_j_* belong to same helix subclass, while in across subclass merging, the neighboring helices *h_i_* and *h_j_* need not belong to the same subclass. The within subclass merging is used to characterize sequence structure relationship in the helix subclasses in terms of overall and position specific propensities [2,5,12] of amino acids in the merged helices. The across subclass merging is used for prediction of sequence based structural variation in helices.

### Sequence based prediction of structural variations

We train a support vector machine (SVM) for automatic prediction of structural variations in the *α*-helix based on its sequences. The helices in the training examples are represented with a set of features derived from their sequences as described in earlier section. The feature set contains overall and position specific amino acid propensities for all the amino acids. Each training example is assigned a label either +1 or -1 based on its subclass.

Let us assume that, we have a training data *D =* {(**x_1_**, *y*_1_), (**x_2_**, *y*_2_),…, (**x_n_**, *y_n_*)} containing *n* vectors, where *i*th vector **x_i_** corresponds to the helix sequence represented with *m* features having label *y_i_* where *y_i_ =* {+1, –1}. The **x_i_** with *y_i_* = 1 are termed as the positive examples and the rest are termed as negative examples. Note here that the vectors in *D* are normalized to length 1. The similarity between two helices **x** and **x′** is calculated as a dot product of **x** and **x′**:(1)

Here (**x**)*_i_* represent *i^th^* feature of the helix sequence (**x**). Based on the training data *D*, SVM learns a hyperplane, which maximizes margin of separation between the positive and negative examples [7]. The SVM is trained using linear kernel function with default parameter settings of SVMLight software [16,17]. The feature weights are obtained from *v* support vectors identified in SVM model. Each support vector *i* has corresponding class label *y_i_* weight *α_i_* and a feature vector **x***_i_*. The weight *w_f_j__* for each feature, *f_j_*, is derived using the following equation(2)

The SVM is tested with 10-fold cross-validation and accuracy is measured in terms of precision, recall and F1 score [7].

## Competing interests

The authors declare that they have no competing interests.

## Authors' contributions

AVT and PW conceived the idea, analyzed the results and wrote the manuscripts. AVT carried out the experiments.
